# Identification of different *ALK* mutations in a pair of neuroblastoma cell lines established at diagnosis and relapse

**DOI:** 10.18632/oncotarget.13541

**Published:** 2016-11-24

**Authors:** Lindi Chen, Angharad Humphreys, Lisa Turnbull, Angela Bellini, Gudrun Schleiermacher, Helen Salwen, Susan L. Cohn, Nick Bown, Deborah A. Tweddle

**Affiliations:** ^1^ Wolfson Childhood Cancer Research Centre, Northern Institute for Cancer Research, Newcastle University, Newcastle upon Tyne, NE1 7RU, United Kingdom; ^2^ Northern Genetics Service, The Newcastle upon Tyne Hospitals NHS Foundation Trust, Institute of Genetic Medicine, Central Parkway, Newcastle upon Tyne, NE1 3BZ, United Kingdom; ^3^ Institut Curie, 26 rue d’Ulm, 75248 Paris cedex 05, France; ^4^ Department of Pediatrics, University of Chicago, Chicago, Illinois 60637, USA

**Keywords:** Neuroblastoma, ALK, R1275L, F1174L, paired cell lines

## Abstract

Anaplastic Lymphoma Kinase (ALK) is a transmembrane receptor kinase that belongs to the insulin receptor superfamily and has previously been shown to play a role in cell proliferation, migration and invasion in neuroblastoma. Activating *ALK* mutations are reported in both hereditary and sporadic neuroblastoma tumours, and several ALK inhibitors are currently under clinical evaluation as novel treatments for neuroblastoma. Overall, mutations at codons F1174, R1275 and F1245 together account for ∼85% of reported *ALK* mutations in neuroblastoma. NBLW and NBLW-R are paired cell lines originally derived from an infant with metastatic *MYCN* amplified Stage IVS (Evans Criteria) neuroblastoma, at diagnosis and relapse, respectively. Using both Sanger and targeted deep sequencing, this study describes the identification of distinct *ALK* mutations in these paired cell lines, including the rare R1275L mutation, which has not previously been reported in a neuroblastoma cell line. Analysis of the sensitivity of NBLW and NBLW-R cells to a panel of ALK inhibitors (TAE-684, Crizotinib, Alectinib and Lorlatinib) revealed differences between the paired cell lines, and overall NBLW-R cells with the F1174L mutation were more resistant to ALK inhibitor induced apoptosis compared with NBLW cells. This pair of cell lines represents a valuable pre-clinical model of clonal evolution of *ALK* mutations associated with neuroblastoma progression.

## INTRODUCTION

Neuroblastoma is an embryonal tumour, originating from progenitor cells of the sympathetic nervous system, and is the most common extra-cranial solid tumour of childhood. In contrast to other childhood cancers, the long-term survival of patients with high-risk neuroblastoma remains poor (< 50%) despite intensive multimodal therapy, with those that survive often suffering from long-term toxicities. Novel targeted therapies which may improve survival while reducing toxicity are under development.

Inhibitors against Anaplastic Lymphoma Kinase (ALK), a transmembrane receptor kinase that belongs to the insulin receptor superfamily, are under clinical evaluation as novel agents to treat neuroblastoma. Originally identified in anaplastic large cell lymphoma as an oncogenic fusion protein with nucleophosmin consequent to a t(2;5)(p23;q35) translocation [1], numerous ALK fusion proteins have since been identified in malignancies including lung, breast, colon and renal cancer, and activating point mutations in non-small cell lung cancer and neuroblastoma (reviewed by [2]). The exact physiological role of ALK remains unclear, however studies have shown that it is preferentially expressed in the developing nervous system with expression levels diminishing postnatally, supporting a role in embryonic nervous system development (reviewed by [2]). The *ALK* gene located on chromosome 2p23, encompasses 29 exons, encoding a 1620 amino acid protein with an extracellular ligand-binding domain, a transmembrane domain, and intracellular juxtamembrane and kinase domains [3]. Activation via ligand binding leads to receptor dimerisation, autophosphorylation, adaptor protein recruitment and subsequent downstream signal transduction through numerous pathways such as RAS/MAPK, PI3K/AKT and JAK/STAT [2, 3].

In neuroblastoma, ALK has been shown to be involved in cell proliferation, migration and invasion and *ALK* mutations have been reported in around 50% of hereditary and 8-10% of sporadic cases, occurring across all risk groups and more frequently at relapse [4–9]. The most common mutation hotspots are located within the kinase domain at codons F1174, R1275 and F1245, which together account for ∼85% of reported *ALK* mutations and result in a constitutively activated protein with *in vitro* transforming capabilities [6]. The co-occurrence of the F1174 *ALK* mutation and *MYCN* amplification has previously been reported, and identifies patients with a particularly poor outcome [6, 8]. In support of this, *in vivo* tissue targeted expression of *ALK^F1174L^* leads to the development of neuroblastoma in transgenic mice, and cooperates with MYCN to accelerate tumour onset with enhanced penetrance and lethality [10, 11]. Previous studies have also reported that both wt and mutant ALK can regulate the transcription of *MYCN* [12], and that *ALK* is a MYCN target gene [9]. Low copy number gains and amplifications of *ALK* have also been reported in neuroblastoma. Almost without exception, *ALK* amplification is accompanied by *MYCN* amplification [6, 8, 13, 14]. In general, *ALK* mutations and amplification are mutually exclusive, however very rare cases of both have been reported [15, 16]. ALK overexpression in the absence of mutation or amplification has also been reported and may have prognostic significance [17].

ALK inhibitors have exhibited anti-tumour activity in preclinical models of neuroblastoma [14, 18], although only modest, responses were observed in a Phase I trial of single agent Crizotinib in paediatric patients [19]. Paediatric Phase 2 studies of Crizotinib monotherapy in patients with *ALK* aberrations (ClinicalTrials.gov; NCT00939770 and NCT02034981), and Phase I evaluation of Crizotinib in combination with existing frontline chemotherapies (NCT01606878) are currently underway. A recent study of *ALK* aberrations in 1,596 diagnostic neuroblastomas showed that different *ALK* mutations confer differential oncogenic potential and sensitivity to Crizotinib, demonstrating the clinical relevance of mutational status for therapeutic stratification of ALK therapies for patients [6]. These observations underline the importance of a robust *ALK* testing strategy for neuroblastoma tumours, and assumptions about the clonal stability of *ALK* mutations may influence whether tumours tested at presentation are re-tested at relapse.

The current study describes the identification of distinct *ALK* mutations using both Sanger and targeted deep sequencing in the paired NBLW and NBLW-R cell lines. The NBLW cell line was established from the primary untreated (right) adrenal tumour of a 6 month old male patient with *MYCN* amplified Stage IVS (Evans Criteria) neuroblastoma with metastasis to the liver [20]. The paired cell line, NBLW-R, was derived post-chemotherapy (4 courses of 70 mg/kg cyclophosphamide and 30 mg/m^2^ daunomycin) approximately 6 months after initial diagnosis from the bone marrow aspirate of the patient at disease relapse with evidence of metastatic disease to the bone and bone marrow and enlarging liver lesions. The patient unfortunately died from the disease < 1 year after initial diagnosis [20]. These paired cell lines representing an interesting model of spatial and clonal/disease evolution are a valuable model for preclinical studies.

## RESULTS AND DISCUSSION

### Array comparative genomic hybridisation (Array CGH) and short tandem repeat (STR) DNA loci analyses

**Figure 1:**
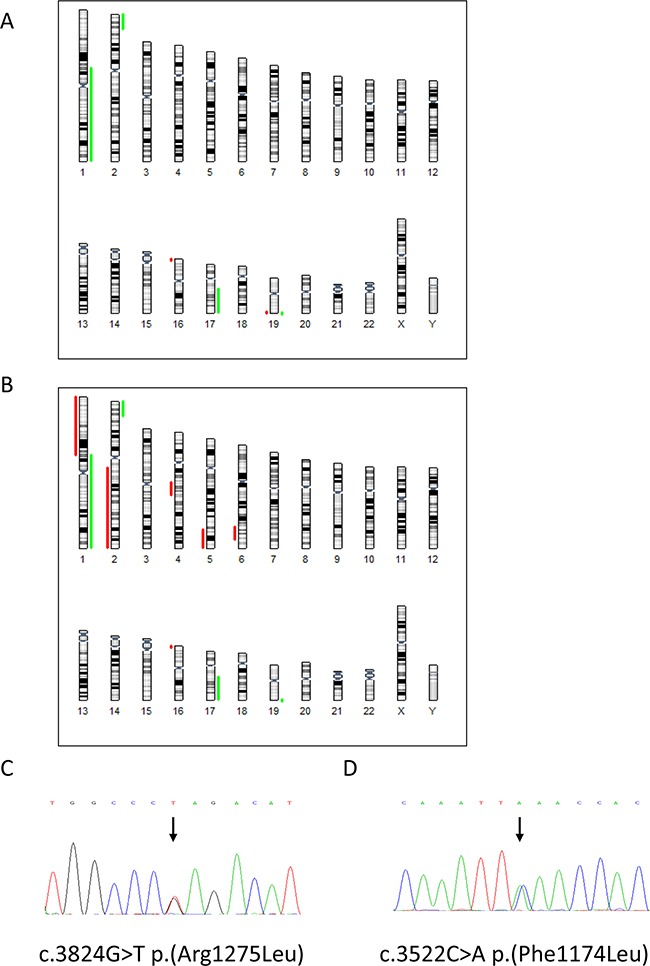
Array CGH results of A NBLW and **B**. NBLW-R cell lines, depicting chromosomal gains in green and losses in red. Chromatograms showing the heterozygous *ALK* mutations in **C**. NBLW and **D**. NBLW-R cells.

The molecular karyotypes of NBLW and NBLW-R cells were determined by array CGH (Figure 1A & 1B) and confirmed that both cell lines were *MYCN* amplified as previously reported [20, 21], and that neither cell line had *ALK* copy number abnormalities. *ALK* was not included in the segmental gains of 2p detected by array CGH, the *ALK* locus being proximal to the 2p breakpoints. A summary of the chromosomal gains and losses detected is shown in Figure 1A & 1B and summarised in Table 1A. This is consistent with both our own karyotype analysis (Supplementary Figure S1) and the previously reported karyotype for the NBLW cell line, and confirms that both cell lines are derived from the same patient [21]. The NBLW-R cell line karyotype is previously unpublished (Supplementary Figure S2), and the present array CGH results demonstrate that in comparison with the NBLW cells (Figure 1A) established at diagnosis, the relapsed NBLW-R cell line (Figure 1B) has several additional regions of chromosomal loss indicative of increased genomic instability, as previously reported in relapsed neuroblastoma [22]. Interestingly, fluorescence *in situ* hybridisation showed that the *MYCN* homogeneously staining regions (HSRs) is present on chromosome 19 in the NBLW cell line, but present on chromosome 9 in the NBLW-R cell line (Supplementary Figure S3). Independent STR genotyping of NBLW and NBLW-R cells confirmed that both cell lines were established from the same patient (Supplementary Figure S4).

**Table 1:** A) Summary of the chromosomal gains and losses in NBLW and NBLW-R cell lines

NBLW	NBLW-R
Gain	
152Mb at 1p21.2q44	152Mb at 1p21.2q44
24 MB at 2p25.3p23.3	24Mb at 2p25.3p23.3
38Mb at 17q21.31q25.3	38Mb at 17q21.31q25.3
1.4Mb at 19q13.43	1.4Mb at 19q13.43
**Loss**	
	96Mb at 1p36.33p21.3
	132Mb at 2q13q37.3
	22Mb at 4q21.21q24
	30Mb at 5q33.1q35.3
	21Mb at 6q23.3q25.3
1.5Mb at 16p13.3	1.5Mb at 16p13.3
0.3Mb at 19q13.43	

B) Base frequencies (mutated allele fractions) at the F1174 and R1275 hotspots in NBLW and NBLW-R cell lines											
Chr position/patient N°	Number of reads	T		G		C		A		Codon change	AA change
Chr2:29432664		%	*P*-Value	%	*P*-Value	%	*P*-Value	%	*P*-Value		
Controls (ALL)	2965617	0.01	NS	99.915	NS	0.001	NS	0.074	NS		
NBLW	273021	50.245	<1E-200	49.731	<1E-200	0.006	NS	0.015	NS	cGa>cTa	R1275L
NBLW-R	291705	0.008	NS	99.946	NS	0.001	NS	0.044	NS	-	-
Chr position/patient N°	Number of reads	T		G		C		A		Codon change	AA change
Chr2:29443695		%	*P*-Value	%	*P*-Value	%	*P*-Value	%	*P*-Value		
Controls (ALL)	2725764	0.01	NS	0.002	NS	99.958	NS	0.029	NS		
NBLW	219604	0.006	NS	0.001	NS	99.961	NS	0.031	NS	-	-
NBLW-R	253575	0.006	NS	0.005	NS	50.389	<1E-200	49.599	<1E-200	ttC>ttA	F1174L

The base corresponding to the reference genome (Human Genome Browser, http://genome.ucsc.edu/ hg19) is indicated at a given coordinate. For a sample to be analysed, the total number of high-quality reads obtained by Hiseq deep sequencing is indicated, and the percentage of reads supporting each base (A, C, G, T) is shown. Values reported for controls are calculated from the total number of reads for germline controls at the given position. The mean base frequencies observed in the control set is also indicated. For each case, the P value refers to the comparison (two-sided Fisher's exact test) of the base frequency observed in the studied sample to that observed in the controls. Abbreviations: NS, not statistically significant; AA, amino acid

### *ALK* mutational screening

Sanger sequencing of exons 20-29 of *ALK* was initially used to determine the *ALK* status of NBLW and NBLW-R cells, and identified a heterozygous R1275L mutation (c.3824G>T; CGA>CTA) in the NBLW cell line established at diagnosis (Figure 1C) and a heterozygous F1174L mutation (c.3522C>A; TTC>TTA) in the NBLW-R cell line establish post-treatment at disease relapse (Figure 1D). Due to the identification of distinct mutations, and because Sanger sequencing is unable to reliably detect mutations present at < 20%, targeted deep sequencing of *ALK* mutation hotspots within exons 23, 24 and 25 was performed using the paired-end Illumina Hiseq2500® procedure as previously described [15] to determine whether the reciprocal mutations were present at a sub-clonal level. Amplicon sequencing (Illumina HiSeq2500) achieved an extremely high depth of coverage (80,000×). The background base variability (error rate) in 10 control samples was 0.017%+/-0.010; thus a base frequency > 0.06% was significantly different from background noise (Fisher's exact test). Consistent with Sanger sequencing, targeted deep sequencing confirmed the R1275L mutation (c.3824G>T; CGA>CTA) detected with a mutated allele fraction of 50.245% (Table 1B). In the NBLW-R cell line, deep sequencing confirmed Sanger sequencing results and detected the F1174L mutation (c.3522C>A; TTC>TTA) with a mutated allele fraction of 49.599% (Table 1B). The deep sequencing data verifies that the reciprocal *ALK* mutations were not present at a low sub-clonal level.

The two mutations identified in NBLW and NBLW-R cells are in line with previous studies which have reported F1174 and R1275 as the commonest sites of *ALK* mutation in neuroblastoma [6]. F1174L mutations have been reported in neuroblastoma cell lines and tumours, and are well characterised, promoting ligand independent autophosphorylation and oncogenic transforming capacity *in vitro* and *in vivo* [6, 8, 18]. F1174L is reported exclusively in somatic cases [3], and often in *MYCN* amplified cases, and is associated with both intrinsic and acquired resistance to Crizotinib [6, 8, 18, 19, 23]. Somatic R1275L mutations have been reported in a few primary neuroblastomas but to our knowledge not yet in cell lines [6, 8, 13, 14, 16, 24–26], and although the conformational and functional consequences are largely unknown, R1275L has been suggested to function through the same mechanism as R1275Q [27].

To our knowledge, this is the first report of 2 distinct *ALK* mutations in cell lines established at diagnosis and relapse from the same patient, and of the R1275L mutation in a neuroblastoma cell line. The presence of two different *ALK* mutations in the same tumour sample has previously been reported [13, 25, 28], and a recent study of *ALK* mutations in paired primary neuroblastoma samples at diagnosis and relapse identified a paired sample which had different *ALK* mutations at diagnosis and relapse but at the same locus and resulted in the same amino acid substitution [7]. The presence of distinct mutations in the NBLW and NBLW-R cell lines represents an interesting pattern of clonal selection, showing elimination of the R1275L clone and relapse with a newly mutant F1174L clonal sideline. Two recent whole-genome/exome sequencing studies of paired neuroblastoma samples at diagnosis and relapse have shed light on the clonal evolution of neuroblastoma and reported that although relapsed samples have a higher incidence of mutations, many mutations which were present at diagnosis are no longer present at relapse [29, 30]. Our findings also support the importance of ALK in neuroblastoma pathogenesis, in particular, the presence of the F1174L mutation in the relapsed NBLW-R cell line is in line with previous reports of the functional cooperation between MYCN and F1174L, representing a particularly aggressive phenotype [6, 8, 10]. It cannot be completely ruled out that the alternative mutations may be present below the detection limit of the method used in this study.

Intra-tumour clonal and spatial heterogeneity may also be a possibility, in that the cell line established from primary tumour material taken at diagnosis contains different clones to the ones which eventually metastasised to bone marrow from which the relapsed cell line was derived. It is also possible that the mutations occurred during the *in vitro* establishment and culture of the cell lines by providing a selective growth advantage. A previous analysis of the CLB-Ba cell line and the patient bone marrow sample from which the cell line was derived, identified the *ALK* mutation in the primary sample at 6.6% and an expansion of the mutation to 32.4% in the cell line [7].

Targeted next generation sequencing (NGS) was performed on NBLW and NBLW-R cells for a panel of 38 genes of established or potential significance in neuroblastoma which was designed in collaboration with the SIOPEN Biology Group (Table 2). In addition to the above *ALK* mutations, a *NF1* (neurofibromin 1) mutation was identified in NBLW cells and a *PTPRD* (protein tyrosine phosphatase, receptor type D) mutation in NBLW-R cells. NF1 is a tumour suppressor and negative regulator of the RAS/MAPK pathway which is downstream of ALK signalling and has previously been shown to be mutated or deleted in primary neuroblastoma, where low *NF1* expression correlated with poor outcome [31]. The absence of the *NF1* mutation in the cell line established at relapse is interesting, as a previous study reported an increased frequency of genetic aberrations of the RAS/MAPK pathway in tumours and cell lines at relapse [29]. PTPRD is a tyrosine phosphatase involved in neuritogenesis and tumour suppressor which has been reported to be inactivated through structural alterations, microdeletions and aberrant splicing [32–34]. Low *PTPRD* expression has been associated with high-risk neuroblastoma, in particular those with *MYCN* amplification [34] and has been reported to function as a tumour suppressor in neuroblastoma by destabilising Aurora Kinase A [35]. The lack of overlap between the gene alterations identified in these cell lines established from the same patient at diagnosis and relapse further highlights an interesting clonal evaluation and is consistent with a previous study which identified altered mutational burden and signatures in primary and relapsed neuroblastoma samples [30].

**Table 2:** Targeted NGS analysis of NBLW and NBLW-R

Cell line	Gene	Chromosome	Alt variant read	Codon change	Amino acid change
**NBLW**	*ALK*	2	41.8%	cGa/cTa	R1275L
	*NF1*	17	98.5%	Gaa/Taa	E91-^1^
**NBLW-R**	*ALK*	2	42.4%	ttC/ttA	F1174L
	*PTPRD*	9	61.6%	cCt/cAt	P711H

Alt, Alternative; ^1^ Stop codon

### ALK mRNA and protein levels in the paired cell lines

**Figure 2:**
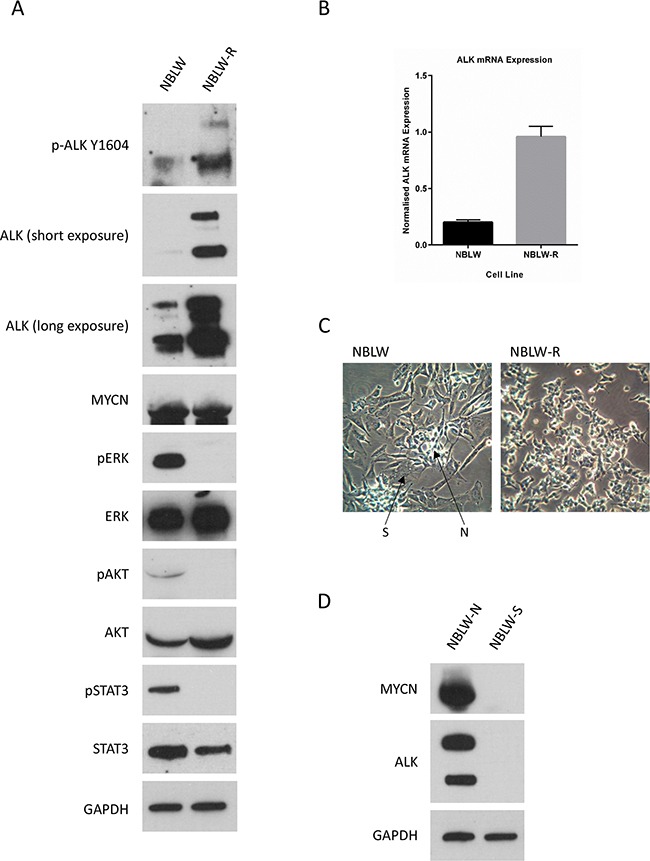
**A**. ALK protein expression and downstream signalling and **B**. *ALK* mRNA in NBLW and NBLW-R cells. **C**. Photomicrographs of the morphological appearance of NBLW and NBLW-R cells. N- and S-type cells are as indicated in the mixed population NBLW cell line. **D**. ALK and MYCN protein expression in NBLW-N and NBLW-S cells.

Analysis of basal expression of ALK and MYCN in the NBLW and NBLW-R cell lines showed that, consistent with their *MYCN* amplified status, both cell lines expressed high levels of MYCN protein (Figure 2A). Compared with NBLW cells, NBLW-R cells expressed higher levels of total and phosphorylated (Y1604) ALK (Figure 2A). Interestingly, however, analysis of downstream signalling through MAPK, AKT and STAT3 pathways showed that NBLW cells expressed higher levels of pERK, pSTAT3 and pAKT compared with NBLW-R cells (Figure 2A). This may be due to the identified loss of *NF1* in NBLW cells. In addition, the higher levels of pERK in NBLW cells compared to NBLW-R cells are consistent with our observations of the greater sensitivity of NBLW cells to MEK inhibitors, Trametinib and MEK162, compared with NBLW-R cells (*data not shown*).

Consistent with ALK protein expression, analysis of *ALK* mRNA levels showed that NBLW-R cells expressed 4.7 fold greater *ALK* mRNA than NBLW cells (Figure 2B), and suggests that the differential expression of ALK between the cell lines is not solely due to post-translational mechanisms such as protein stability. The transcriptional control of *ALK* has not been fully elucidated; however *ALK* has previously been reported to be a direct target gene of both MYCN and PHOX2B [9] but, as both cell lines show comparable MYCN expression levels, it is unlikely to be due to MYCN mediated transcription of *ALK* (Figure 2A). Alternatively, the different morphological phenotype of N- and S-type mixed population NBLW cells (50% N:50% S; Figure 2C) versus N-type NBLW-R cells may be influencing ALK expression, as a previous study observed lower levels of ALK expression in S-type versus N-type neuroblastoma cells [14]. Consistent with this, separation of NBLW cells into NBLW-N and NBLW-S showed that NBLW-N cells expressed higher levels of ALK, as well as MYCN, compared with NBLW-S cells (Figure 2D), despite the presence of the R1275L *ALK* mutation in both cell types (*data not shown*). It is also possible that the difference is a consequence of the different *ALK* mutations detected in NBLW and NBLW-R cells, as the R1275L mutation present in NBLW cells is not well characterised. Although previous studies in neuroblastoma primary tumours and cell lines have established that *ALK* mutant cases expressed higher levels of *ALK* mRNA and/or protein compared to wt cases, no differences were reported between different ALK mutations [14, 36].

### Sensitivity of cell lines to ALK inhibitors

Studies have previously reported that the presence of the F1174L mutation confers relative resistance to Crizotinib but not to TAE-684, of which an analogue, LDK378 (Ceritinib), is presently in paediatric clinical trials (ClinicalTrials.gov; NCT01742286) [6, 18]. Second generation ALK inhibitor, Alectinib, and third generation, PF-06463922 (Lorlatinib), have also been reported to be effective against F1174L mutants [37, 38]. The sensitivity of NBLW and NBLW-R cells to Crizotinib, TAE-684, Alectinib and PF-06463922 were determined using XTT cell proliferation assays. Both cell lines were found to be sensitive to Crizotinib, TAE-684 and Alectinib mediated growth inhibition, with TAE-684 observed to be >10 times more potent than Crizotinib and Alectinib (Table 3). No significant differences were observed between Crizotinib GI_50_ values of NBLW and NBLW-R cells (Table 3), however, NBLW-R cells were significantly more sensitive to TAE-684 and Alectinib mediated growth inhibition compared to NBLW cells. In addition, while NBLW-R cells were found to be sensitive to the third generation dual ALK/ROS1 inhibitor, PF-06463922, 50% growth inhibition could not be achieved even at the highest tested concentration of 10μM in NBLW cells (Table 3). The latter may be due to differences in the selectivity of PF-06463922 towards the different mutations present in NBLW and NBLW-R cells, in particular as little is known about the R1275L mutation. In addition, aberrations in other pathways which influence sensitivity to ALK inhibitors may be present, such as ROS1. The above data highlight interesting differences between different ALK inhibitors and their therapeutic potential in the treatment of *ALK* mutant neuroblastoma.

**Table 3:** 72h GI_50_ values for ALK inhibitors in NBLW and NBLW-R cells

Cell Line	NBLW	NBLW-R	*P*-value
**Crizotinib**	584.0 ± 41.6 nM	494.2 ± 43.5 nM	0.22
**TAE-684**	38.5 ± 3.9 nM	30.2 ± 5.3 nM	0.02
**Alectinib**	488.5 ± 49.1 nM	307.3 ± 18.3 nM	0.02
**PF-06463922 (Lorlatinib)**	> 10 μM	169.9 ± 20.6 nM	< 0.0001

Data represent n ≥ 3 ± SEM.

**Figure 3:**
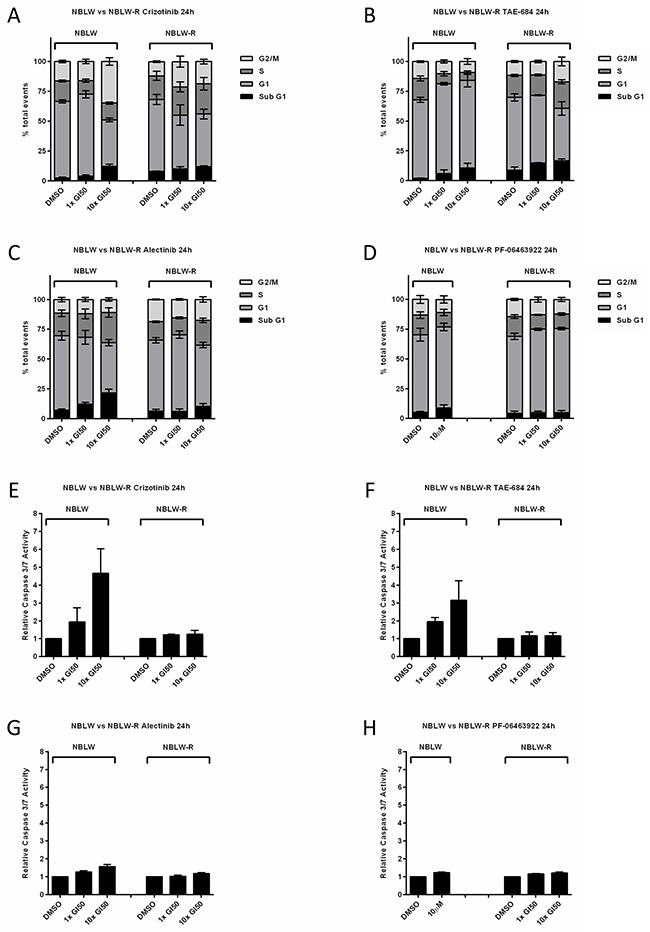
Sub-G1 and cell cycle phase distribution of NBLW and NBLW-R cells treated for 24 hours with DMSO, 1× or 10× their respective GI50 concentrations of ALK inhibitors A Crizotinib, **B**. TAE-684, **C**. Alectinib and **D**. PF-06463922*. Caspase 3/7 activity of NBLW and NBLW-R cells treated for 24 hours with DMSO, 1× or 10× their respective GI_50_ concentrations of **E**. Crizotinib, **F**. TAE-684, **G**. Alectinib and **H**. PF-06463922*. *NBLW cells were treated with 10 μM PF-06463922 only. Caspase 3/7 data are expressed as fold change relative to DMSO control. All data are the average of at least 3 independent experiments and error bars represent SEM.

Functional analysis using flow cytometry and caspase 3/7 assays determined the effect of ALK inhibitors Crizotinib, TAE-684, Alectinib and PF-06463922 on NBLW and NBLW-R cells (Figure 3). Overall, the results show that Crizotinib, TAE-684 and PF-06463922 induced a G_1_ cell cycle arrest, whereas Crizotinib, TAE-684 and Alectinib induced dose-dependent apoptosis in NBLW cells (Figure 3). In NBLW-R cells, Crizotinib and TAE-684 led to an accumulation of cells in G_2_/M, and Alectinib and PF-06463922 induced a G_1_ cell cycle arrest, however all tested ALK inhibitors had minimal effects on apoptosis (Figure 3). The latter is consistent with the more aggressive nature of the relapsed cell line, and possibly result from aberrations in pathways regulating apoptosis acquired during disease relapse. In conclusion, this pair of cell lines represents an interesting pre-clinical model of clonal evolution and neuroblastoma tumourigenesis, and underscore the need for *ALK* analysis at both diagnosis and relapse to detect additional or different *ALK* aberrations which may affect response to ALK inhibitors.

## MATERIALS AND METHODS

### Cell culture and DNA extraction

*MYCN* amplified human neuroblastoma cell lines, NBLW and NBLW-R were cultured in RPMI 1640 (Sigma, Dorset, England) supplemented with 10% v/v Foetal Calf Serum (Gibco/Life Technologies Ltd, Paisley, UK). Photomicrographs (×20) were captured using a VisiCam® digital camera and analyser software (VWR International Ltd, Lutterworth, UK). DNA was extracted using the DNeasy Blood and Tissue Kit (Qiagen, Manchester, UK) according to the manufacturer's instructions, and quantified using the Nanodrop (Thermo Scientific, Waltham, MA USA). Cell line authentication was conducted using the AuthentiFiler™ PCR Amplification Kit (Applied Biosystems/Life Technologies Ltd) and Promega PowerPlex® 16 HS System according to manufacturer's protocols.

### Array CGH and targeted NGS

Array CGH was performed using Agilent whole genome 80×60K oligo array (ISCA version 2.0) and Illumina BlueFuse Multi v3.3 analysis software with Genome Build GRCh37, providing a resolution of below 0.25 Mb. Sanger sequencing was performed using standard methods and primer sequences are available on request. Illumina HiSeq2500 sequencing was used to achieve a very high depth of coverage within *ALK* exons 23, 24 and 25 containing the F1174, F1245 and R1275 hotspots, respectively. DNA was amplified via a two-step PCR approach, the second step consisting of addition of sample-specific barcodes for targeted resequencing in a single experiment. Targeted NGS was performed on a panel of 38 genes of established or potential significance in neuroblastoma, designed in collaboration with the SIOPEN Biology Group:- *NRAS*, *MDM4*, *MYCN*, *ALK*, *IDH1*, *PIK3CA*, *PDGFRA*, *TERT*, *FGFR4*, *CDK6*, *BRAF*, *FGFR1*, *MYC*, *CDKN2A*, *CDKN2B*, *PTCH1, TSC1, PTPRD, PTEN*, *HRAS*, *CCND1*, *ATM*, *KRAS*, *CDK4*, *MDM2*, *PTPN11*, *MAP2K1*, *TP53*, *NF1*, *ERBB2*, *MAP2K2*, *ATRX*, *ARID1A*, *ARID1B*, *PDE6G*, *TENM2*, *MAP3K13* and *PHOX2B*. The Illumina Truseq custom amplicon kit was used for NGS library preparation, and sequenced using the Illumina NextSeq 550. Analysis was carried out using Illumina TruSeq Amplicon BaseSpace Amplicon software, Illumina Variant Studio software and Alamut® Visual (Interactive Biosoftware, Rouen, France).

### Cell proliferation assays

Cell proliferation was determined using XTT assays and GI_50_ concentrations calculated as previously described [39]. Crizotinib, TAE-684, Alectinib and PF-06463922 were obtained from Selleck Chemicals (Munich, Germany) and diluted in DMSO (Sigma). All statistical tests were performed using GraphPad Prism v6.0 software.

### Quantitative reverse transcription PCR (qRT-PCR), protein analysis, flow cytometry and Caspase 3/7 assays

RNA was extracted using RNeasy Mini kit (Qiagen) and quantified using the Nanodrop. qRT-PCR was performed using inventoried TaqMan Gene Expression Assays (Applied Biosystems). RNA expression values were normalised to GAPDH and all experiments were performed in triplicate. Cell lysates were harvested using PhosphoSafe lysis buffer (Merck Millipore Ltd, Feltham, UK) and Western blotting carried out as previously described [39]. Primary antibodies used were p ALK (Tyr-1604 and Tyr-1282/83), ALK, p ERK, ERK, pAKT, AKT, pSTAT3 (Cell Signaling, Leiden, The Netherlands), STAT3 (R&D Systems) MYCN and GAPDH (Santa Cruz Biotechnology, Heidelberg, Germany), all at 1:1000. Flow cytometry and Caspase 3/7 assays were performed as previously described [39].

## SUPPLEMENTARY FIGURES


