# Supplementary material for: Identification of different ALK mutations in a pair of neuroblastoma cell lines established at diagnosis and relapse
Source: Oncotarget. 2016 Nov 24;7(52):87301–11. doi: 10.18632/oncotarget.13541 (PMC5349989; doi:10.18632/oncotarget.13541)
Supplement: Supplementary file 1 [file oncotarget-07-87301-s001.pdf]

Identification of different *ALK* mutations in a pair of neuroblastoma cell lines established at diagnosis and relapse

SUPPLEMENTARY FIGURES

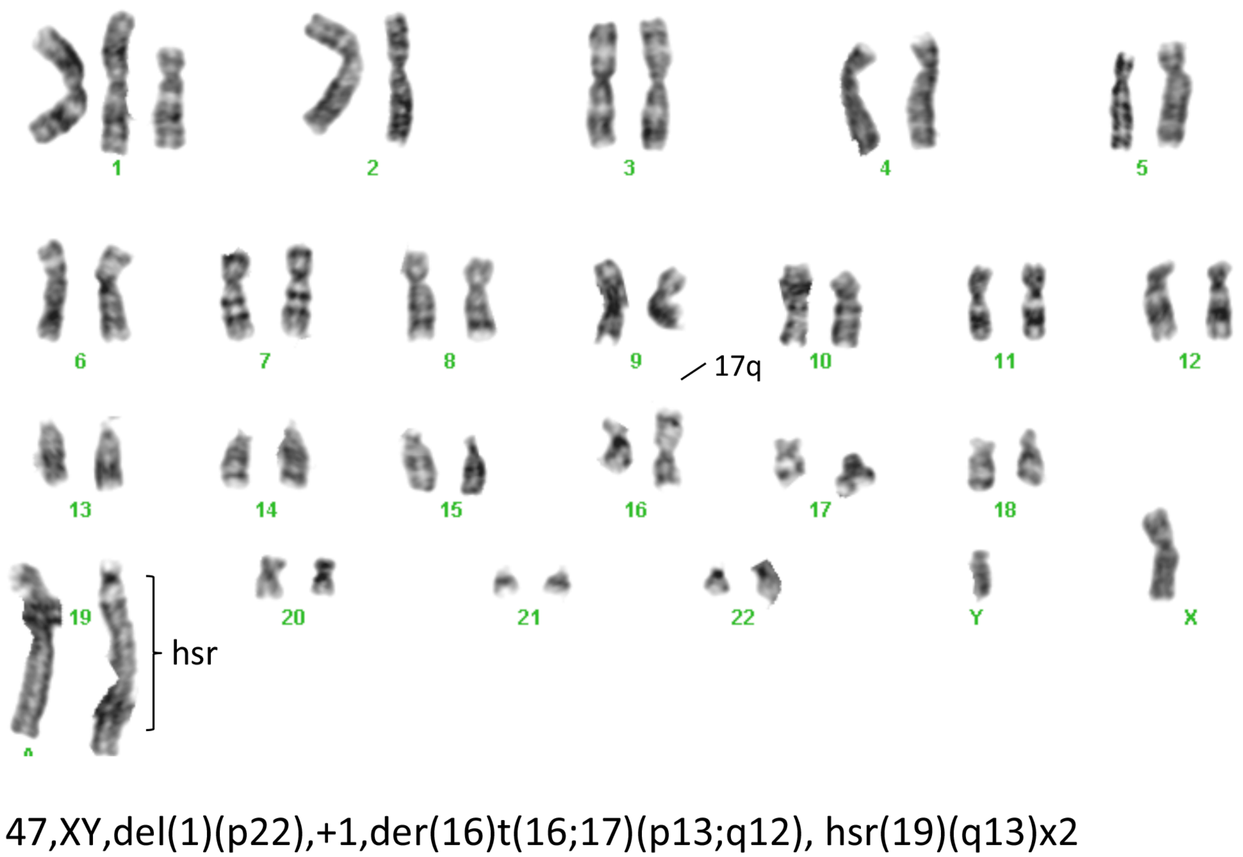

Supplementary Figure S1: Cytogenetic G-banding analysis of the karyotype of NBLW cell line using previously described methods (Tweddle et al, Cancer Res. 2001 Jan 1;61(1):8-13.).

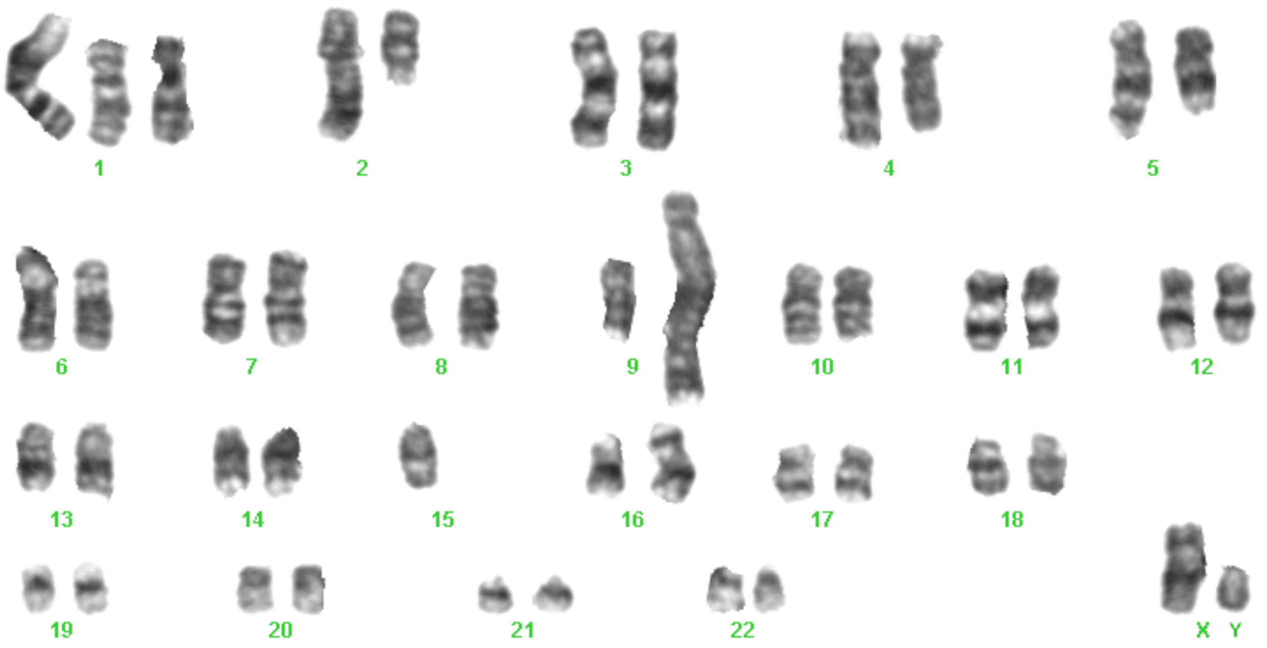

47,XY,del(1)(p22)x2,+1,del(2)(q13),del(4)(q2?q2?),del(5)(q33),
?del(6)(q?),hsr(9)(q?),der(16)t(16;17)(p13;q12)

Supplementary Figure S2: Cytogenetic G-banding analysis of the karyotype of NBLW-R cell line using previously described methods (Tweddle et al, Cancer Res. 2001 Jan 1;61(1):8-13.).

NBLW
HSRs <u>not</u> in chromosome 9

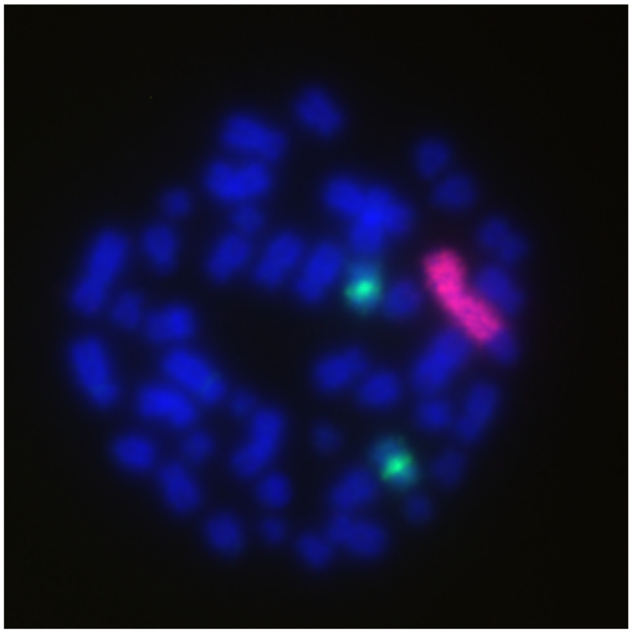

NBLW-R
HSRs in chromosome 9

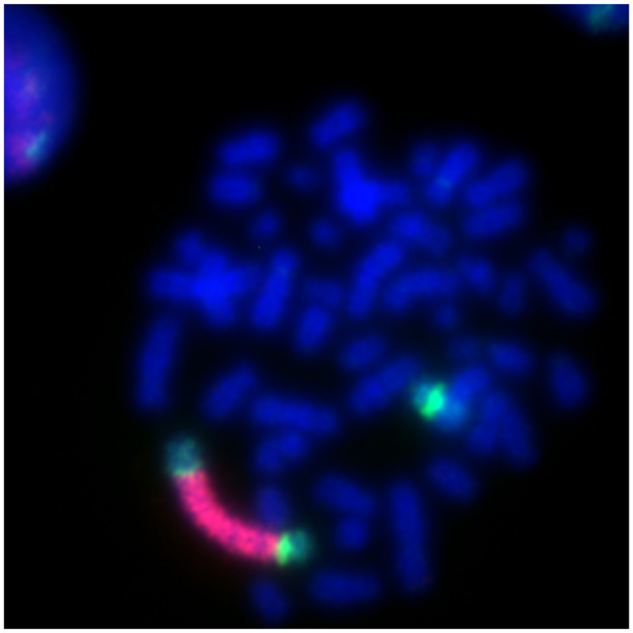

Supplementary Figure S3: Fluorescence *in situ* hybridisation using the MYCN probe (Red; Vysis) and chromosome 9 whole chromosome paint (green; Vysis) with previously described methods (Tweddle et al, Cancer Res. 2001 Jan 1;61(1):8-13.) confirms location of *MYCN* homogeneously staining regions (HSRs).

A)

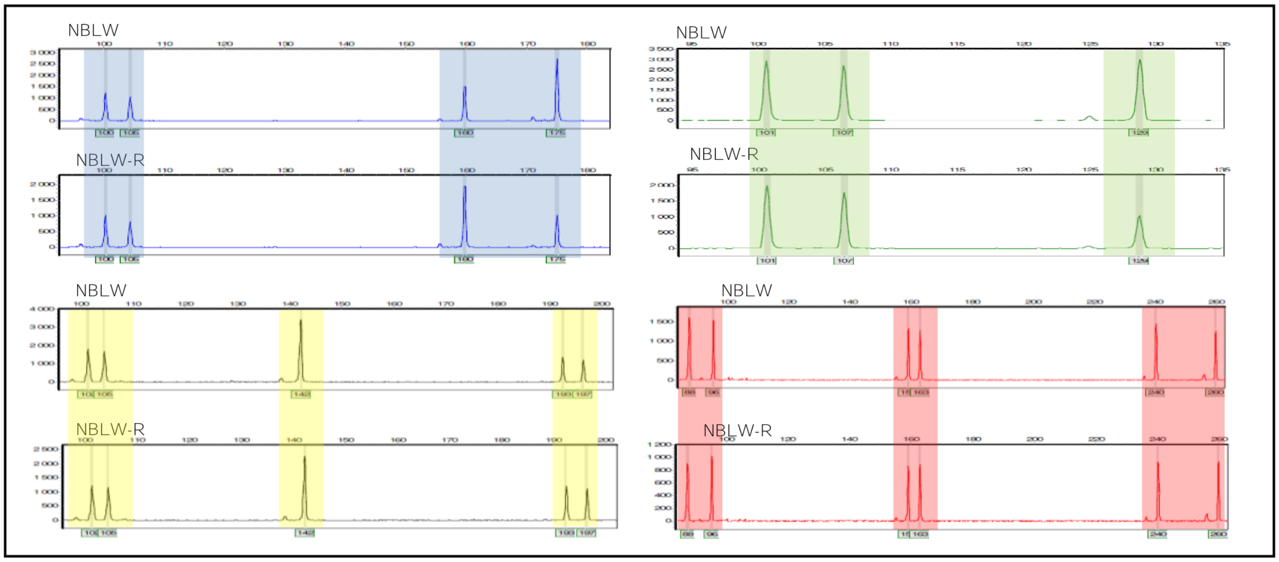

B)

Loci	Cell Line	
	NBLW	NBLW-R
Amelogenin	X – Y	X – Y
D3S1358	14 – 16	14 – 16
THO1	7 – 8	7 – 8
D21S11	28 – 30	28 – 30
D18S51	14 – 14	14 – 14
PentaE	13 – 14	13 – 14
D5S818	13 – 14	13 – 13
D13S317	8 – 11	8 – 11
D7S820	11 – 12	11 – 12
D16S539	11 – 12	11 – 12
CSF1PO	12 – 12	12 – 12
PentaD	13 – 13	13 – 13
vWA	16 – 17	16 – 17
D8S1179	14 – 14	14 – 14
TPOX	8 – 10	8 – 10
FGA	22 – 24	22 – 23

Supplementary Figure S4: Independent STR genotyping of NBLW and NBLW-R cells compared using A. Applied Biosystems AuthentiFiler™ PCR Amplification Kit and **B.** Promega PowerPlex® 16 HS System and confirmed that both cell lines were established from the same patient.
